# Supplementary material for: Detection of neutralizing antibodies against multiple SARS-CoV-2 strains in dried blood spots using cell-free PCR
Source: Nat Commun. 2022 Jul 21;13:4212. doi: 10.1038/s41467-022-31796-1 (PMC9302227; doi:10.1038/s41467-022-31796-1)
Supplement: Supplementary file 1 — Supplementary Information [file 41467_2022_31796_MOESM1_ESM.pdf]

SUPPLEMENTARY INFORMATION

Detection of neutralizing antibodies against multiple SARS-CoV-2 strains in dried blood spots using cell-free PCR

Supplementary Table 1. Intra-assay variation of SONIA neutralization test. Patient samples were tested in replicates to evaluate the intra-assay variations.

	Positive Sample 1			Positive Sample 2			Negative Sample		
	Delta	Alpha	WT	Delta	Alpha	WT	Delta	Alpha	WT
Number of samples	7	7	7	7	7	7	7	7	7
Average	5.32	7.04	5.04	4.58	4.89	4.43	0.54	0.03	0.41
Standard Deviation	0.19	0.24	0.20	0.42	0.64	0.47	0.25	0.43	0.29
%CV	3.57%	3.52%	4.00%	9.12%	13.17%	10.56%	N/A	N/A	N/A
Positive Rate	7/7	7/7	7/7	7/7	7/7	7/7	0/7	0/7	0/7

Supplementary Table 2. Inter-assay variation of SONIA neutralization test. Patient samples were tested in replicates to evaluate the inter-assay variations.

	Positive Sample 1			Positive Sample 2			Negative Sample		
	Delta	Alpha	WT	Delta	Alpha	WT	Delta	Alpha	WT
Number of samples	21	21	21	21	21	21	21	21	21
Average	5.08	6.74	5.04	4.25	5.37	4.42	0.08	-0.28	-0.03
Standard Deviation	0.51	0.60	0.33	0.58	0.90	0.71	0.64	0.51	0.59
%CV	9.96%	8.86	6.64	13.63%	16.72	16.17	N/A	N/A	N/A

		%	%		%	%			
Positive Rate	21/21	21/21	21/21	21/21	21/21	21/21	0/21	0/21	0/21

Supplementary Table 3. Summer shipping simulation condition. To evaluate the impact of shipping on neutralizing antibodies in dried blood spot formats, we conducted temperature cycling study to mimic shipping under summer and winter time. The temperature cycling profiles were consistent with the guidance from the FDA neutralizing antibody testing template.

Temperature	Cycle Period	Cycle Period Hours	Total Time Hours
40°C	1	8	8
22°C	2	4	12
40°C	3	2	14
30°C	4	36	50
40°C	5	6	56

Supplementary Table 4. Winter shipping simulation condition. To evaluate the sample stability during the winter transport process, the specimens were stored in the following temperature for the specified period and subjected to SONIA analysis.

Temperature	Cycle Period	Cycle Period Hours	Total Time Hours
-20°C	1	8	8
22°C	2	4	12
-20°C	3	2	14
4°C	4	36	50
-20°C	5	6	56

Supplementary Table 5. Shipping simulation result for neutralizing antibody (wild type, alpha and delta). The results of sample stability analysis after various shipping condition

simulations were provided below.

Sample Group	Test Point	N	Positive (%)
Negative	T=0	10	0 (0)
	Summer	10	0 (0)
	Winter	10	0 (0)
Low Positive 1	T=0	20	20 (100)
	Summer	20	20 (100)
	Winter	20	20 (100)
Low Positive 2	T=0	10	10 (100)
	Summer	10	10 (100)
	Winter	10	10 (100)

Supplementary Table 6. DBS card drying time and neutralizing antibody signals (for wild type, alpha and delta). Samples dropped onto DBS cards were allowed to dry for 8, 30, 60 and 240 minutes (the recommended drying time is 240 minutes). The samples were then closed and placed into the sample biohazard bag with desiccant.

Drying time	Positive Samples	Neutralizing antibody PPV	Negative Samples	Neutralizing antibody NPV
8 min	4/4	100%	4/4	100%
30 min	4/4	100%	4/4	100%
60 min	4/4	100%	4/4	100%
240 min	4/4	100%	4/4	100%
Summary	16/16	100%	16/16	100%

Supplementary Table 7. Alcohol pad drying time and neutralizing antibody signals (wild type, alpha and delta). To simulate a user wiping the subject's finger prior to obtaining the blood sample, pipette tips used to apply blood to the DBS paper were wiped with alcohol pads and allowed to dry for 4, 15 and 30 sec. After pipetting onto the DBS paper, the blood spots were allowed to dry per the collection instruction.

Alcohol drying time on finger	Positive Samples	Neutralizing antibody PPV	Negative Samples	Neutralizing antibody NPV
4 sec	4/4	100%	4/4	100%
15 sec	4/4	100%	4/4	100%
30 sec	4/4	100%	4/4	100%
Summary	12/12	100%	12/12	100%

Supplementary Table 8. DBS stability after freeze-thaw for neutralizing antibodies against delta strain. DBS samples were tested by multiplex SONIA assay for delta neutralizing antibodies after various freeze-thaw cycles.

Freeze-thaw /Delta	Positive Sample 1	Determination	Positive Sample 2	Determination	Negative Samples	Determination
2 cycles	5.40	Positive	4.90	Positive	0.04	Negative
5 cycles	5.57	Positive	4.92	Positive	-0.35	Negative
8 cycles	5.58	Positive	4.59	Positive	-0.14	Negative

Supplementary Table 9. DBS stability after freeze-thaw for neutralizing antibodies against wild type strain. DBS samples were tested by multiplex SONIA assay for wild type neutralizing antibodies after various freeze-thaw cycles.

Freeze-thaw /WT	Positive Sample 1	Determination	Positive Sample 2	Determination	Negative Samples	Determination
2 cycles	6.01	Positive	5.73	Positive	-0.32	Negative
5 cycles	5.39	Positive	5.01	Positive	-0.40	Negative
8 cycles	5.65	Positive	5.41	Positive	-0.67	Negative

Supplementary Table 10. DBS stability after freeze-thaw for neutralizing antibodies against alpha strain. DBS samples were tested by multiplex SONIA assay for alpha neutralizing antibodies after various freeze-thaw cycles.

Freeze-thaw /Alpha	Positive Sample 1	Determination	Positive Sample 2	Determination	Negative Samples	Determination
2 cycles	7.34	Positive	5.77	Positive	0.16	Negative
5 cycles	7.38	Positive	5.70	Positive	-0.11	Negative
8 cycles	7.12	Positive	5.68	Positive	-0.14	Negative

Supplementary Table 11. DBS eluent stability after freeze-thaw for neutralizing antibodies against delta strain. DBS eluents were tested by multiplex SONIA assay for delta neutralizing antibodies after various freeze-thaw cycles.

Freeze-tha	Positiv	Determinatio	Positiv	Determinatio	Negativ	Determinatio

w Eluent /Delta	e Sample 1	n	e Sample 2	n	e Samples	n
1 cycles	5.14	Positive	4.62	Positive	0.45	Negative
2 cycles	5.17	Positive	3.56	Positive	0.54	Negative
3 cycles	5.21	Positive	4.22	Positive	0.89	Negative
4 cycles	5.22	Positive	4.42	Positive	0.44	Negative
5 cycles	5.56	Positive	4.30	Positive	0.97	Negative

Supplementary Table 12. DBS eluent stability after freeze-thaw for neutralizing antibodies against wild type strain. DBS eluents were tested by multiplex SONIA assay for wild type neutralizing antibodies after various freeze-thaw cycles.

Freeze-thaw Eluent /WT	Positive Sample 1	Determination	Positive Sample 2	Determination	Negative Sample	Determination
1 cycles	5.59	Positive	5.28	Positive	0.25	Negative
2 cycles	5.49	Positive	3.69	Positive	0.24	Negative
3 cycles	5.40	Positive	4.11	Positive	0.74	Negative
4 cycles	5.41	Positive	4.43	Positive	0.14	Negative
5 cycles	5.79	Positive	4.41	Positive	0.99	Negative

Supplementary Table 13. DBS eluent stability after freeze-thaw for neutralizing antibodies against alpha strain. DBS eluents were tested by multiplex SONIA assay for alpha neutralizing antibodies after various freeze-thaw cycles.

Freeze-thaw Eluent /Alpha	Positive Sample 1	Determination	Positive Sample 2	Determination	Negative Sample	Determination
1 cycles	6.59	Positive	5.51	Positive	0.53	Negative
2 cycles	6.48	Positive	5.09	Positive	0.66	Negative
3 cycles	7.19	Positive	5.51	Positive	1.52	Negative
4 cycles	6.31	Positive	5.15	Positive	0.76	Negative
5 cycles	7.0	Positive	5.09	Positive	1.36	Negative

Supplementary Table 14. Comparison of SONIA with mainstay COVID-19 neutralizing test. Various COVID-19 neutralizing antibodies were compared based on their technical

attributes.

Assay Comparison	PRNT	Pseudo virus assay	Surrogate ELISA (e.g. Genscript)[1]	SONIA
BSL3 required	Yes	No	No	No
Turnaround time	Weeks	Days to weeks	1-2 hr (hand on time included)	2-3 hr (hand on time included)
Fully validated for dried blood spots (DBS)	No	No	No	Yes
Multiplex	No	No	No	Yes
Specialized instruments for readout	No	No	No	No

Supplementary Table 15. DNA sequences and primers. The sequences of DNA barcodes and primers used in the study is provided below.

WT	CTTTCAGAGGTCAGGTGTATTCACGATGAGACTGGATGAA
Alpha	GGATCACTCCAACTAGACTATCACGATGAGACTGGATGAA
Delta	ATCAGGTCTGGAAGATTACGTCACGATGAGACTGGATGAA
ACE2	TCACGGTAGCATAAGGTGCACAGTAACGATGTATCCACAG
WT Primers Forward	CTTTCAGAGGTCAGGTGTAT
Alpha Primers Forward	GGATCACTCCAACTAGACTA
Delta Primers Forwrad	ATCAGGTCTGGAAGATTACG
ACE2 Primer Reverse	CTGTGGATACATCGTTACTG

Supplementary Figure 1. Representative image of protein-DNA conjugates. Silver staining gel image of protein and protein-DNA conjugates. Lane 1 and 2: unconjugated spike protein S1 subunit in replicates. Lane 3 and Lane 4: Spike protein S1 subunit with DNA conjugated in replicates. A clear mass shift was observed, affirming successful DNA conjugation. The protein-DNA conjugate has protein concentration of 2753nM (determined

by BCA) assay and DNA concentration of 2984nM (determined by UV-Vis at 260nm). Thus, the DNA/protein conjugation ratio is 1.1. This experiment was conducted for a total of two times.

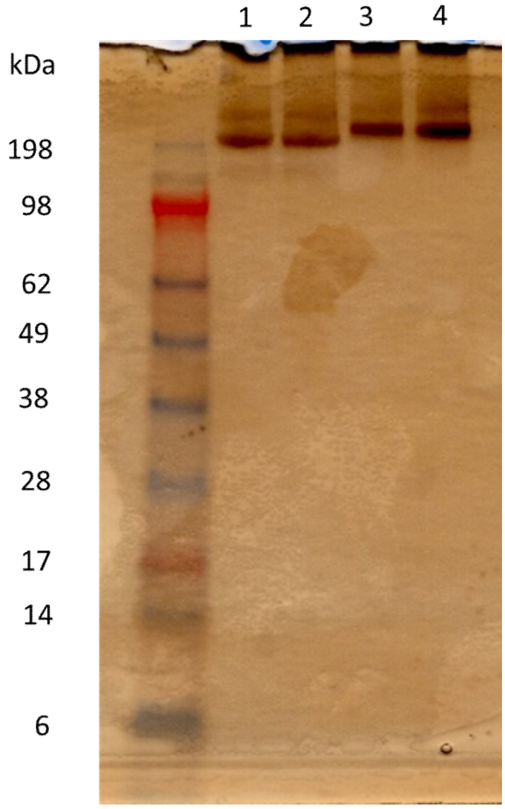

REFERENCES

1. Tan, C.W., et al. A SARS-CoV-2 surrogate virus neutralization test based on antibody-mediated blockage of ACE2-spike protein-protein interaction. Nat Biotechnol 38(9):1073-1078. (2020).
