# Supplementary material for: Detection of neutralizing antibodies against multiple SARS-CoV-2 strains in dried blood spots using cell-free PCR
Source: Nat Commun. 2022 Jul 21;13:4212. doi: 10.1038/s41467-022-31796-1 (PMC9302227; doi:10.1038/s41467-022-31796-1)
Supplement: Supplementary file 3 — Reporting Summary [file 41467_2022_31796_MOESM3_ESM.pdf]

nature portfolio

Corresponding author(s): Cheng-ting Tsai

Last updated by author(s): 2022/05/16

Reporting Summary

Nature Portfolio wishes to improve the reproducibility of the work that we publish. This form provides structure for consistency and transparency in reporting. For further information on Nature Portfolio policies, see our Editorial Policies and the Editorial Policy Checklist.

Statistics

For all statistical analyses, confirm that the following items are present in the figure legend, table legend, main text, or Methods section.

n/a	Confirmed	
☐	☒	The exact sample size (*n*) for each experimental group/condition, given as a discrete number and unit of measurement
☐	☒	A statement on whether measurements were taken from distinct samples or whether the same sample was measured repeatedly
☐	☒	The statistical test(s) used AND whether they are one- or two-sided<br>*Only common tests should be described solely by name; describe more complex techniques in the Methods section.*
☒	☐	A description of all covariates tested
☐	☒	A description of any assumptions or corrections, such as tests of normality and adjustment for multiple comparisons
☐	☒	A full description of the statistical parameters including central tendency (e.g. means) or other basic estimates (e.g. regression coefficient) AND variation (e.g. standard deviation) or associated estimates of uncertainty (e.g. confidence intervals)
☐	☒	For null hypothesis testing, the test statistic (e.g. *F*, *t*, *r*) with confidence intervals, effect sizes, degrees of freedom and *P* value noted<br>*Give P values as exact values whenever suitable.*
☒	☐	For Bayesian analysis, information on the choice of priors and Markov chain Monte Carlo settings
☐	☒	For hierarchical and complex designs, identification of the appropriate level for tests and full reporting of outcomes
☐	☒	Estimates of effect sizes (e.g. Cohen's *d*, Pearson's *r*), indicating how they were calculated

Our web collection on statistics for biologists contains articles on many of the points above.

Software and code

Policy information about availability of computer code

Data collection	Data is collected on Bio-Rad CFX96 and CFX384.
Data analysis	PRISM (version 8.1.1) and XLSTAT software (version 2018.1) were used for data and statistical analysis.

For manuscripts utilizing custom algorithms or software that are central to the research but not yet described in published literature, software must be made available to editors and reviewers. We strongly encourage code deposition in a community repository (e.g. GitHub). See the Nature Portfolio guidelines for submitting code & software for further information.

Data

Policy information about availability of data

All manuscripts must include a data availability statement. This statement should provide the following information, where applicable:

- Accession codes, unique identifiers, or web links for publicly available datasets
- A description of any restrictions on data availability
- For clinical datasets or third party data, please ensure that the statement adheres to our policy

All relevant data are within the paper and its supporting information/source data file. Source data are provided with this paper.

Field-specific reporting

Life sciences study design

All studies must disclose on these points even when the disclosure is negative.

Sample size	In the serum study, we included 146 COVID19 serum and 43 non-COVID serum. In the kinetic study, we have a total of 28 serum samples. In the DBS study 1, we have a total of 27 COVID-19 DBS, 20 control DBS, and 9 vaccinated DBS. In the DBS study 2, we have a total of 40 vaccinated DBS, 80 healthy controls DBS, and 40 Lyme DBS. The number of specimens were chosen based on the FDA EUA guidance document for COVID-19 neutralization assay.
Data exclusions	None
Replication	All of the samples above were run as a single replicate. Authors have conducted additional efforts to evaluate the reproducibility of the data using intra-assay and inter-assay study. Results were provided in Supplementary Table 1 and 2.
Randomization	The samples were analyzed in a randomized manner.
Blinding	The testing staff were also blinded to the disease group of the samples.

Reporting for specific materials, systems and methods

We require information from authors about some types of materials, experimental systems and methods used in many studies. Here, indicate whether each material, system or method listed is relevant to your study. If you are not sure if a list item applies to your research, read the appropriate section before selecting a response.

Materials & experimental systems

n/a	Involved in the study	
☒	☐	Antibodies
☒	☐	Eukaryotic cell lines
☒	☐	Palaeontology and archaeology
☒	☐	Animals and other organisms
☐	☒	Human research participants
☒	☐	Clinical data
☒	☐	Dual use research of concern

Methods

n/a	Involved in the study	
☒	☐	ChIP-seq
☒	☐	Flow cytometry
☒	☐	MRI-based neuroimaging

Human research participants

Policy information about studies involving human research participants

Population characteristics	A total of 393 de-identified human specimens were used in this manuscript. The COVID-19 patients were determined to be COVID-19 infected if positive by RT-PCR for COVID-19 RNA.
Recruitment	All participants provided informed consents including future research use authorization.
Ethics oversight	Blood specimens from SARS-CoV-2 RNA-positive individuals were obtained from various sources: Leftover serum samples originally submitted for COVID-19 serological testing were obtained from Oregon Health Sciences University Hospital (OHSU), the California Department of Public Health, and Mayo Clinic. These were sourced from discarded clinical laboratory specimens exempted from informed consent and IRB approval under condition of patient anonymity. Three plasma samples were obtained from COVID-19 convalescent outpatient plasma donors of the University Hospital Basel, Switzerland with informed consent. These donors were screened in compliance with Swiss regulations on blood donation and approved as plasma donors in accordance with national regulations. Convalescent COVID-19 patients and vaccinated individuals were recruited to donate matched serum samples and finger-prick blood for the study with informed consent under Enable Biosciences IRB #20180015 (approved by Western IRB). Serum samples collected prior to the outbreak from blood donors and individuals with other viral or bacterial infection were purchased from commercial biobanks. All specimens collected outside of Enable Biosciences clinical network were received as de-identified specimens.

Note that full information on the approval of the study protocol must also be provided in the manuscript.
